# Progress in Medicine

**Published:** 1897-12-11

**Authors:** 


### Progress in Medicine.

### DISEASES OF THE LUNGS.

(*Continued from page* 174).

**Phthisis.**—Stubbert[8] has examined the chest in cases of pulmonary tuberculosis by means of the Roentgen rays and fluorescent screen, and finds that by this method not only valuable corroborative evidence can be obtained of results arrived at by auscultation and percussion, but also in some instances isolated foci of infection can be found, not recognisable by ordinary methods. Seventy-three patients were inspected; and, in order to make diagnosis more sure, the examination by the fluorescent screen, and by percussion and auscultation, were carried out by different observers. He finds that slight haziness indicates the beginning of tuberculous infiltration, whilst decided shadows indicate consolidation. Circumscribed spots of bright reflex, surrounded by narrow dark shadow rings or in the midst of an area of dense shadow, indicate cavities. Intense darkness, especially in the lower part of the lung, indicates old pleuritic thickenings over consolidated lung tissue.

The detection of a cavity in the lungs by the ordinary methods of percussion and auscultation, is often a matter of not merely scientific interest, but practical importance, for it may often render a probable diagnosis certain, especially when the disease is quiet and the physical signs doubtful. Maguire[9] is inclined to lay most stress on the following points: (1) A cavernous percussion note; the cavity itself causes hyper-resonance or percussion, but the layer of consolidated lung tissue over it gives a dull note, and it is by this combination of tympanicity and dulness that we get the cavernous percussion note which is so characteristic. (2) Cavernous breathing; the essence of which, as compared with bronchial breathing, is its hollowness. (3) Cavernous râles; which are produced by disturbance of fluid in the cavity. These are not only metallic in character but are "hollow," with a peculiar resonance or echo. An exaggeration of these cavernous râles is a splashing râle, best heard when the patient coughs, and so disturbs the fluid. (4) "Tussive resonance;" when a healthy person coughs, the sound is not reinforced, but if a cavity be present it may be so loud as to be almost painful to the listening ear. (5) Post-tussive suction sound; when a cavity has been compressed by the expiratory effort of a cough, and is then suddenly released, its walls expand, and a certain amount of air is sucked back, producing a characteristic suction sound, accompanied as a rule by crepitations. Maguire also says that whispering pectoriloquy is certainly the least valuable of all the signs to be heard over a pulmonary cavity, for it may be heard well over pneumonic consolidation, and sometimes over an empyema; hence it cannot be relied upon.

The fact that heart disease has a most important influence on the occurrence and development of tubercle in the lungs has long been known, but its nature and mechanism is a matter of dispute. Graham[10] finds that mitral disease and pulmonary tuberculosis rarely co-exist, whilst pulmonary tuberculosis is a very frequent sequel of pulmonary stenosis. The presence of mitral disease acts as a preventative of tuberculosis, especially when the vital powers are at or near their normal standard; whereas when, from age or bad habits, the vital powers are on the decline, the process is often as rapid as in ordinary cases. This prophylactic agency of mitral lesions is seen particularly well in those who have a strong hereditary tendency to the disease, and whose surroundings are of a decidedly unhealthy character. The mechanism of this prophylaxis is probably to be sought in several factors, especially in the fact that mitral disease produces a passive congestion of the lungs, and this, in its turn, an increased amount of chest measurement, especially at the apices, thus expanding the alveoli. Also there is an increase in the involuntary muscular tissue, which enables the patient, more especially by coughing, to expel foreign matter from the alveoli and bronchi.

**Treatment of Phthisis: Koch's New Tuberculin.**—Koch[11] has brought out a new preparation which may be used in the treatment of phthisis and other tubercular lesions. He prefaces his article by saying that there are two kinds of immunity—a toxic and a bacterial. Thus Behring and Kitasato have prepared a tetanus anti-toxin, which neutralises the poisons produced by the tetanus bacilli which vegetate unhindered in the immunised body. The defect of this immunity is that, though great, it lasts only a certain time. On the other hand, Pfeiffer has shown that a bacterial immunity against typhoid and cholera can be produced —a state in which the animal is protected against the living bacteria, which, if injected, rapidly die, but is not protected against the poison produced by them, for if this be injected the animals die. The ideal immunisation is against both of these dangers, against the germs and their poisons. The prospect of immunity in tuberculosis seems unfavourable, yet in miliary and experimental tuberculosis it exists in some measure because at a certain stage of the disease the germs disappear. The difficulty is to produce immunity early enough to save the individual. Koch has tried all kinds of methods to bring this about. All efforts to cause bacilli to be absorbed have failed. Dead bacilli injected caused abscess, or death from secondary effects, as when introduced into the peritoneum. Tubercle bacilli have a fatty acid capsule which so protects them from outside influences that they are not absorbed. This capsule was destroyed by repeatedly grinding young virulent cultures in an agate mortar until they were thoroughly broken up. The mass was then mixed with water and centrifugalised. The lower layers of the fluid so obtained were found to be completely absorbable and non-abscess forming. With this preparation guinea-pigs can be completely immunised against infection with virulent cultures of tubercle bacilli. Koch recommends that this new tuberculin, or R. tuberculin, should be used in the treatment of tuberculosis, in doses beginning with 1-500th milligramme and increased gradually to 20 m.g. Cure of tuberculous animals can only be effected provided treatment is begun early, and this applies as well to people, in whom injections must be early, before infection becomes mixed.

This new preparation has been used by a number of physicians with variable results. Some amount of improvement has been recorded in various tubercular lesions by Slawyk,[12] Wörner,[12] Seeligmann,[12] Bussenius,[12] Schultze,[12] and Sandwith,[13] though untoward results have sometimes occurred; for instance, severe febrile reaction with collapse after doses as small as 1-4,000th milligramme.

[8] New York Medical Recerd, May 22, 1897. [9] Clinical Journal, June 9, 1897. [10] Montreal Medical Journal, Sep. 1896. [11] Medical Week, April 16, 1897. [12] Deut. Med. Woch., July 22, 1897. [13] Lancet, Sep. 4, 1897.

### DISEASES OF THE DIGESTIVE ORGANS.

(*Continued from page* 173.)

**Ulcer of the Dnodenum.**—Algazzi[42] reports three cases in patients suffering from lead poisoning, two of which caused perforation, and he thinks that similar lesions may at times be the source of the colicky pains, since lead causes intense constriction of the small vessels with endarteritis. Dunn,[43] at the Clinical Society, related a successful laparotomy for a perforated ulcer of this kind. While at work the patient was attacked with pain, nausea, and faintness. Tympanites and loss of the liver dulness led to the abdomen being opened, when the perforation was found and sewn up. After some subsequent difficulties were overcome complete recovery took place. Bland Sutton held that many of the subjects of these ulcers were spirit drinkers, and bad subjects for operation. Salol has been found united into a calculus in the stomach or intestines. C. R. Marshall[44] mentions one case where the concretion weighed a gramme, and was vomited after attacks of severe colic, apparently caused by its presence, while others were passed per rectum. A similar case was observed by Brossard, where several calculi were passed; and Girode found a concretion of salol in the stomach of a patient who died from cholera two days after taking the drug. Marshall believes that when the temperature is high (42 C.), salol melts in the stomach and then solidifies into these masses, after which it is slowly, if at all, acted on by the secretions. It ought, therefore, to be given rubbed up with some powder, or in emulsion, to avoid its forming these masses.

**Dysentery.**—Bosc and Vedel[45] find intravenous saline injections of great value in dysentery, which in France is, they think, due to an infection of the colon bacillus. They direct that from ten to eighteen hundred cubic centimetres of normal saline solution should be slowly njected at an early stage of the disease, to lead to a sustained general reaction and altered local conditions. Besides diluting the blood, and causing a great excretion of toxins, the injections seem to act almost as an immunising serum. Sargo[46] had a patient suffering for 11 months from amœbic dysentery of sporadic origin in Vienna, where it is very rare. A cat inoculated from the stools developed hæmorrhagic dysentery, and a second cat was infected from the first after the amœbæ had disappeared. When they were killed some months later the lower part of the small intestine was found inflamed, with glandular swellings and ulcers, but there were no amœbæ. The function of these organisms has been disputed, and these results do not make the part they play any clearer. C. E. Lockwood,[47] however, reports a severe case where the amœbæ were found in the stools and treated by injections of quinine for five days, the strength being increased from 1 in 5,000 to 1 in 1,500, and the amount finally raised to four pints, with a little starch and opium beforehand. Complete recovery took place, with the disappearance of all the parasites from the stools at the same time. In a paper on the treatment of intestinal diseases Turck[48] advocates the use of small bran cakes for dyspepsia and intestinal putrefaction, and gives the bran to young children suspended in milk. He finds that it not only aids the digestive fluids, but renders the intestinal walls less septic. He also employs in certain cases for irrigation a hot-water bag with double tube, and a spray of oil of cloves and cinnamon, the latter especially in treatment of dilated colon. Finally, he claims that by his gyromele he has succeeded in passing a tube into the colon itself. This has long been said to be impossible with a flexible tube owing to the difficulty of the sigmoid flexure, and even with his revolving tube success cannot always be attained.

The detection of fluid in the peritoneum is sometimes easier, as Professor Bard showed, if fluctuation is sought when the patient is sitting up, and the impulse is produced from the loins towards the front of the abdomen instead of from side to side. However, this fluctuation has been also found when there was only a stricture of the intestines without any ascites,[49] the distended upper part of the bowels transmitting a wave much as fluid free in the abdomen would. Mathieu,[50] in speaking of membranous colitis, insists that the constipation must be prevented without using irritative purgatives. He prefers castor oil, or cascara with magnesia. Enemata of marsh mallows, or salicylates, are useful, but boric acid or naphtholates are irritating. If dysenteric attacks come on, nitrate of silver injections, one in five thousand, may be employed. The intestinal congestion may be influenced by hydrastis or hammamelis. The nervous state of these patients should be carefully treated, and the diet, of course, regulated with much care if any success is to be obtained.

[42] Med. Bulletin, July. [43] Med. Rec., March 13. [44] Brit. Med. Jour., July 10. [45] Presse Med., June 23. [46] Amer. Jour. Med. Sc., Aug. [47] Med. Rec., April 3. [48] New York Med. Jour., March 13. [49] Med. Week, June 11. [50] Med. Week, July 2.

